# Reduced Intravenous Vancomycin Use and Cost Savings in a Pediatric Hemodialysis Unit: A Quality Report

**DOI:** 10.1097/pq9.0000000000000846

**Published:** 2025-11-11

**Authors:** Shrea Goswami, Neha Pottanat

**Affiliations:** From the * Department of Palliative Medicine, Montefiore Medical Center, Bronx, N.Y.; † Albert Einstein College of Medicine, Bronx, N.Y.; ‡ Department of Pediatric Nephrology, Riley Children’s Hospital, Indianapolis, Ind.; § Indiana University, Indianapolis, Ind.

## Abstract

**Introduction:**

Intravenous (IV) vancomycin is used in patients receiving chronic hemodialysis (HD) for the treatment of central venous catheter–related infections. Excessive use of IV vancomycin leads to high costs and an increase in multidrug-resistant organisms. Our primary aim was to reduce IV vancomycin use for suspected central venous catheter–related infections in our pediatric chronic HD unit by 50% within 1 year. Our secondary aim was to reduce the total cost of IV vancomycin use by 50%.

**Methods:**

With a quality improvement framework, the key interventions include (1) provider education on antibiotic stewardship, related to IV vancomycin and its associated risks; (2) identification of alternative topical/enteral antibiotics; (3) development and implementation of an exit-site scoring tool; and (4) development of a treatment-based algorithm to standardize choice of antibiotic related to exit-site score.

**Results:**

We used a statistical process control chart to demonstrate that IV vancomycin use declined from an average of 24 doses per 1,000 patient-days in the preintervention period (August 1, 2018, to August 31, 2019) to 5.1 vancomycin doses per 1,000 patient-days in the postintervention period (September 1, 2019, to July 31, 2020). Additionally, the IV vancomycin cost decreased from $81,297 per 1,000 patient-days during the preintervention period to $14,053 per 1,000 patient-days during the postintervention period.

**Conclusions:**

A stepwise incorporation of interventions, including education, the development of a novel exit-site scoring tool, and a treatment-based algorithm, resulted in a 78% decrease in IV vancomycin use in our pediatric chronic HD unit within 1 year and an 82% decrease in associated costs.

## INTRODUCTION

A tunneled central venous catheter (CVC) is a common long-term vascular access used in children with end-stage renal disease (ESRD) who undergo chronic hemodialysis (HD). Despite the Kidney Disease Outcomes Quality Initiative guidelines recommending the use of arteriovenous access for long-term use in the United States, 85.6% of children aged 13–17 years use CVCs to initiate HD.^1,2^ Barriers to placement of arteriovenous access include patient size limitations, imminent timing of transplantation, and individual preferences.^3,4^

The prevalence of CVC-related bloodstream infection (CVC-BSI) research in children receiving chronic HD is limited. The Standardizing Care to Improve Outcomes in Pediatric End-Stage Kidney Disease Collaborative reported a CVC-BSI rate of 0.95 per 100 patient-months (~0.32 per 1000 patient-days) in the United States.^4,5^ Meanwhile, the United States Renal Data System (USRDS) reported an all-cause infection hospitalization rate of 0.51 admissions per patient-year in pediatric patients receiving HD.^2^

Broad-spectrum antibiotic use, particularly intravenous (IV) vancomycin, is prevalent in patients undergoing HD due to the risk of methicillin-resistant *Staphylococcus aureus* (MRSA) infection associated with CVCs.^6^ The prevalence of pediatric MRSA has been 1.7 per 100,000 children since 2010.^7–10^ There is limited literature describing antimicrobial practice patterns and stewardship in pediatric HD units.

The Centers for Disease Control and Prevention guidelines recommend aseptic technique, including the use of topical antibiotics, when accessing a tunneled CVC. However, there is a lack of standard diagnostic criteria for a CVC exit-site infection (ESI) or tunnel infection (TI). This lack of standard criteria creates an opportunity for the liberal use of broad-spectrum IV antibiotics. We hypothesized that creating a standardized exit-site scoring tool and treatment algorithm would decrease excessive IV vancomycin use.

Our primary aim was to reduce the use of IV vancomycin in our pediatric chronic HD unit to treat suspected CVC-related infections by 50% (<12 vancomycin doses per 1,000 patient-days) within 1 year. Our secondary aim was to reduce the total cost of IV vancomycin use by 50%.

## METHODS

Our pediatric HD unit has 9 beds, accommodating 12–16 patients who receive HD 3–5 times per week. There are 17 dialysis nurses, a dialysis nurse manager, a dialysis quality coordinator, and a unit secretary who organizes the morning and afternoon shifts of HD treatments. The unit is a small, close-knit ecosystem where frequent interactions ensure that any medical errors are quickly identified and immediately corrected. We identified a high rate of IV vancomycin use in our children receiving HD with tunneled CVCs, specifically relating to the use of prophylactic IV vancomycin for nonocclusive dressing over the CVC exit site. Using the quality improvement (QI) methodology, we defined an objective criterion for assessing a tunneled CVC exit site. We identified alternative topical and enteral antibiotic treatment options for CVC-related ESIs and TIs.

### Planning of the Study

We included children between the ages of 1 and 20 years receiving chronic HD with CVC access. The study was not characterized as human subjects research and was not under the institutional review board’s oversight. The key members of the QI team included 2 dialysis-registered nurses (RNs)—1 identified as the “RN infection champion”—a hospital infection prevention team member, a QI coordinator, a pharmacist, an advanced practice provider (APP), and 2 pediatric nephrology physicians (1 serving as the team leader).

### Planning of Key Interventions

**Fig. 1.**
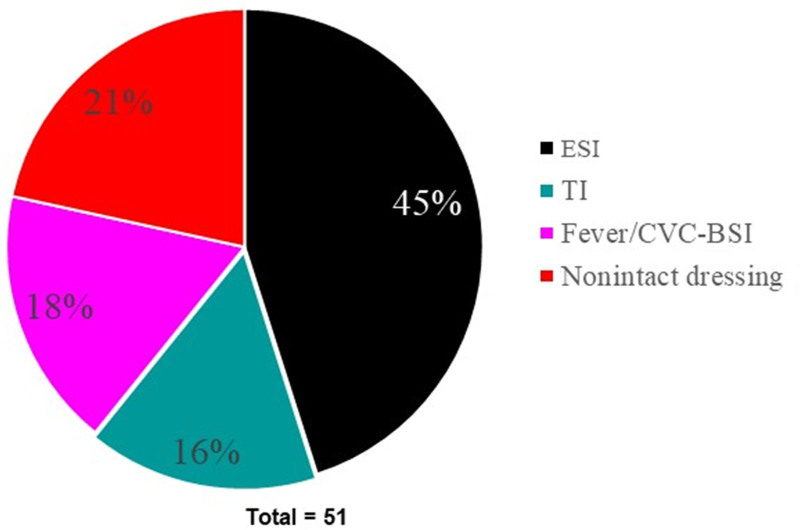
Indications of IV vancomycin use before intervention.

The IV vancomycin rate was 24 vancomycin doses per 1,000 patient-days during the preintervention period (August 1, 2018, to August 31, 2019). The CVC-BSI rate (defined as a positive blood culture at the time of, or within 48 hours of, developing fever/signs of sepsis) was 0.46 per 1,000 patient-days. Chart audit revealed that common reasons for prescribing vancomycin included fever (≥38 °C) with or without positive blood culture, signs of ESI/TI, or the presence of a nonintact CVC dressing (Fig. 1). Of note, our HD unit does not routinely use IV vancomycin lock therapy for prevention of CVC-BSI.

### Key Interventions

**Fig. 2.**
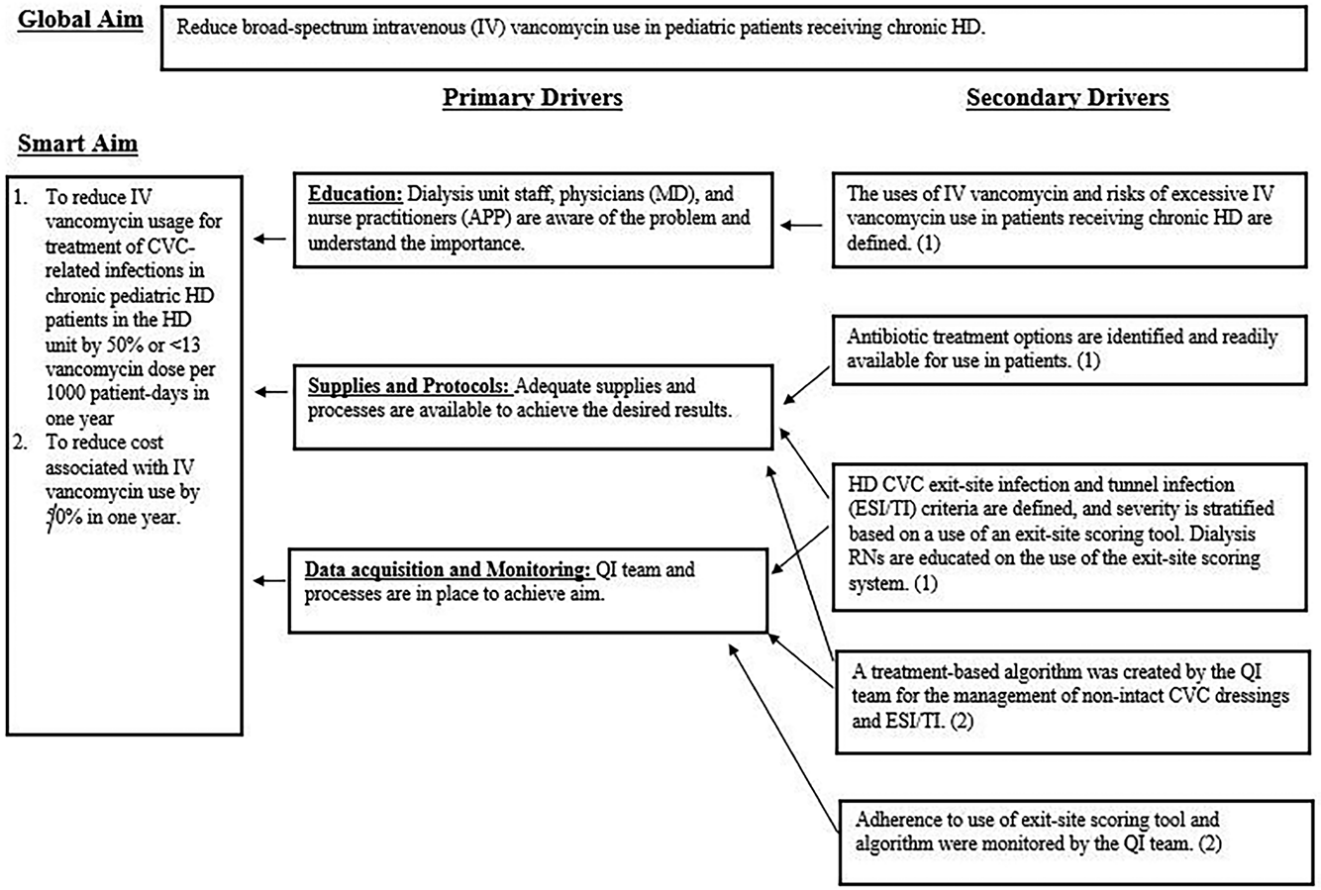
Key driver diagram for vancomycin reduction in a pediatric HD unit. (1) Secondary drivers are directed at the standardization of workflow. (2) Secondary drivers focus on developing error-proof systems.

The Plan-Do-Study-Act cycle methodology guided our implementation of our key interventions. Additionally, a key driver diagram (Fig. 2) guided the development of these interventions. The following describes each step.

#### Plan

We recognized the overuse of IV vancomycin in our pediatric HD unit. The primary objectives were to reduce IV vancomycin prescriptions by 50% and decrease associated costs by 50%.

##### Identification of Alternative Antibiotics and Availability.

In collaboration with our local antibiogram and infection prevention team, we identified topical mupirocin and enteral trimethoprim-sulfamethoxazole as effective alternatives for prophylactic treatment against Gram-positive bacteria, including MRSA, which commonly cause ESIs. We confirmed that both antibiotics were already part of our hospital formulary, with no risk of medication shortage.

#### Do (Interventions)

##### Staff and Provider Education

The QI team leader delivered educational presentations for the dialysis team, describing current prescribing practice and reiterating complications associated with excessive IV vancomycin use in patients receiving chronic HD (ie, development of antimicrobial-resistant organisms, *Clostridium difficile* infections, and prolonged patient treatment time, as vancomycin is administered after completion of HD). The team leader reviewed indications for IV vancomycin use (fever with a source, CVC-BSI/bacteremia, and ESI/TI that are not easily eradicated) and alternative initial topical/enteral antibiotics for ESI/TI.

##### Adaptation and Implementation of the Exit-Site Scoring System

The score (Table 1) was adapted from the peritoneal dialysis (PD) catheter site assessment according to the International Society of Peritoneal Dialysis guidelines.^11,12^ Their guidelines are a valid model because PD catheters and permanent HD CVC lines have tunnels that exit at the skin and require similar exit-site care to prevent infections. The RN infection champion educated the dialysis RNs regarding the exit-site scoring assessment tool during 2 nursing huddles, held 1 month apart. She emphasized the importance of using it routinely during weekly CVC dressing changes and whenever the exit-site dressing was noted to be nonocclusive or soiled. The QI coordinator distributed copies of the exit-site scoring tool, which was accessible for consultation in the dialysis unit. The dialysis RNs were instructed to use the tool beginning in August 2019 after completing their education and documenting its use in the electronic medical record (EMR).

**Table 1.** Exit-Site Scoring System

	0 Point	1 Point	2 Points
Swelling	No	Exit only (<0.5 cm)	Including part of or the entire tunnel (>0.5 cm)
Crust	No	<0.5 cm	>0.5 cm
Redness	No	<0.5 cm	>0.5 cm
Pain	No	Slight	Severe
Discharge	No	Serous	Purulent

Modified from Schaefer F, Klaus G, Müller-Wiefel DE, et al. Intermittent versus continuous intraperitoneal glycopeptide/ceftazidime treatment in children with peritoneal dialysis-associated peritonitis. The Mid-European Pediatric Peritoneal Dialysis Study Group (MEPPS). *J Am Soc Nephrol*. 1999;10:136–145; and Warady BA, Bakkaloglu S, Newland J, et al. Consensus guidelines for the prevention and treatment of catheter-related infections and peritonitis in pediatric patients receiving peritoneal dialysis: 2012 update. *Perit Dial Int.* 2012;32 (Suppl 2):S32–S86.

##### Development and Implementation of a Treatment-based Algorithm

**Fig. 3.**
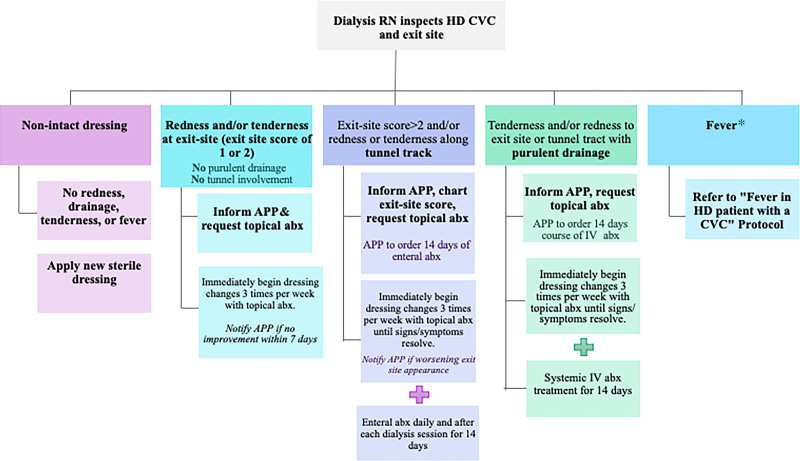
Treatment-based algorithm for patients with signs/symptoms of infection related to CVC. abx, antibiotic; *Fever defined as ≥38.0 °C.

Based on an RN assessment of the exit site, a treatment-based algorithm was developed to determine the next steps (Fig. 3). This algorithm was created after reviewing the existing literature and national consensus guidelines.^13,14,15^ The nephrologists educated the dialysis team on using the algorithm; copies were shared and posted in the dialysis unit. Notably, our previously established HD unit protocol was to collect 1 set of blood cultures from both lumens of the CVC when patients presented with fever or signs of sepsis.

#### Study

The QI coordinator monitored adherence to the new exit-site scoring tool by performing weekly audits using the EMR for the first 3 months and then monthly thereafter. The RN infection champion, coordinator, or QI team leader provided repeated reminders if deviations were found. The dialysis coordinator recorded the number of IV vancomycin doses, infusion time, and quantity of vancomycin administered during both the pre- and postintervention periods.

#### Act

Based on the data and audit findings, we continually adjusted our protocols to achieve our objectives. We implemented these interventions between September 1, 2019, and July 31, 2020.

## MEASURES AND ANALYSIS

### Outcome Measures and Analysis

The primary outcome measure was the vancomycin doses per 1,000 patient-days between September 1, 2019, and July 31, 2020. The dialysis APP and QI coordinator used a statistical process control (SPC) chart to track antibiotic data. The QI team collected the total number of IV vancomycin doses for all causes. The target was less than 12 vancomycin doses per 1000 patient-days. The upper control limit and lower control limit were within 3 SDs of the process mean. SPC chart rules were applied to differentiate special versus common cause variation.

Furthermore, data for a 1-year sustainment period were collected between August 1, 2020, and July 31, 2021. The secondary outcome was cost reduction. The QI team determined the total cost savings both before and after the interventions. The pharmacist provided the price of the vancomycin dose per gram, as well as the associated administrative fees and infusion-related charges.

### Process Measures and Analysis

The QI team documented each exit-site assessment and the resultant intervention (topical, enteral, or IV antibiotics) between August 1, 2018, and July 31, 2021. The QI coordinator audited the use of the exit-site scoring system and the algorithm on a weekly basis for the first 3 months, then reduced the frequency to monthly. The reduction in audit frequency was due to dialysis providers becoming more adherent to using the exit-site tool and treatment-based algorithm; therefore, frequent audits were deemed less necessary.

### Balancing Measures and Analysis

Our balancing measure was the rate of CVC-BSIs during the preintervention period (August 1, 2018, to August 31, 2019) and the postintervention period (September 1, 2019, to July 31, 2020). An increase in CVC-BSI, associated with a recently diagnosed ESI/TI or episode of nonintact dressing, would indicate that the reduced use of prophylactic IV vancomycin was leading to patient harm.^15^ The QI coordinator monitored the CVC-BSI episodes, which were calculated as the CVC-BSI rate per 1,000 patient-days (or catheter-days) between the pre- and postintervention periods.

### Statistical Analysis

The team collected instances of vancomycin use and documentation of ESI, TI, and CVC-BSI between August 1, 2018, and July 31, 2021, in a Microsoft Excel spreadsheet. The SPC charts were created using the Intermountain Healthcare Delivery Institute Advanced Training Program’s QI tools and Microsoft Excel, version 2018.^16^ We chose a U chart to best represent our discrete data over time, given the patient volume that varies each month.^17^

### Cost Analysis

The pharmacist provided the actual purchase price of vancomycin from the pharmacy’s purchasing system. The administrative fee was based on a time-based estimate for each pharmacist, whereas the infusion cost was determined by the hourly nursing rate and standard supplies required per infusion. The QI coordinator collected data on the total grams of vancomycin used and infusion time for each patient, which enabled the calculation of the total drug cost. The estimated drug cost of IV vancomycin was $14.60 per gram, including a $3.00administrative fee for each dose. The infusion cost was $2,461.00 for the initial 90 minutes and an additional $257.00 for any infusion exceeding 90 minutes. We analyzed the total cost reduction by adjusting it per 1,000 patient-days and inflation-adjusted it to 2024–2025 using the Consumer Price Index for both the pre- and postintervention periods.

## RESULTS

### Primary Outcome

**Fig. 4.**
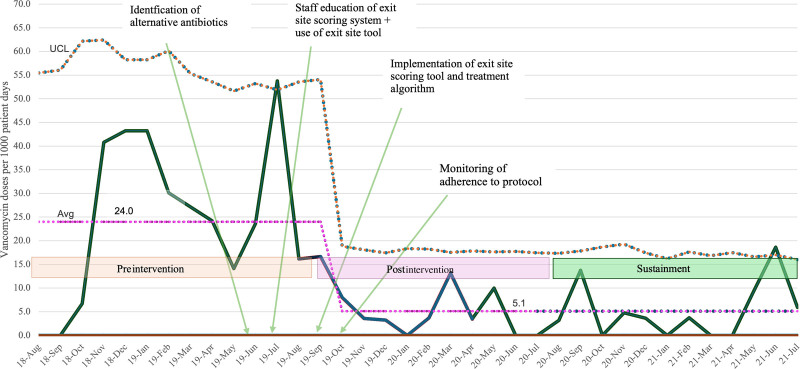
Vancomycin doses per 1,000 patient-days—U chart. IV vancomycin (dose per 1,000 patient-days) use in a tertiary children’s hospital HD unit between August 1, 2018, and July 31, 2021. Avg, average line; UCL, upper control limit.

During the 12-month baseline period (August 1, 2018, to August 31, 2019), the average vancomycin use was 24 doses per 1,000 patient-days. After implementing the 4 interventions in July 2019, the number of vancomycin doses per 1,000 patient-days decreased to 8. With an emphasis on the use of the treatment-based algorithm and monitoring adherence to the algorithm, there was a further decrease in vancomycin doses to 3.6 doses per 1,000 patient-days from September 2019 to December 2019 (Fig. 4). This resulted in an 80% reduction in vancomycin use in the first 6 months. The average vancomycin use decreased to 5.1 doses per 1,000 patient-days in the postintervention period (September 1, 2019, to July 31, 2020).

The special cause events in the preintervention period include 1 point above the upper control limit. During the intervention period, there was a downward shift below the average of 24 doses per 1,000 patient-days between September 2019 and February 2020. During the sustainment period, a downward trend was observed, except for increases in September 2020 and June 2021 (Fig. 4), which the QI team investigated.

### Secondary Outcome

The total cost incurred from IV vancomycin use during the preintervention period (August 1, 2018, to August 31, 2019) was $81,297 per 1,000 patient-days compared with $14,053 per 1,000 patient-days in the postintervention period (September 1, 2019, to July 31, 2020). The total cost savings for IV vancomycin and drug administration were $67,244 per 1000 patient-days, representing an 82% cost reduction.

### Process Measures

Between August 1, 2019, and December 31, 2019, there were 4 instances of documentation of the exit-site scoring system during nonroutine dressing changes. In contrast, between January 1, 2020, and July 31, 2020, there were 23 instances (an 82% increase in documentation). The increase in documentation resulted from repeated reminders provided to dialysis RNs, as documenting the use of the exit-site scoring tool in the EMR was not part of their usual workflow. In addition, there was an increase in topical and enteral antibiotic use (mupirocin, n = 4; trimethoprim-sulfamethoxazole, n = 3), and changing a nonintact dressing without administering IV or oral antibiotics became a more widely accepted practice (n = 26), which was not observed in 2019. The use of the treatment protocol algorithm was not monitored.

### Balancing Measure

One patient had a CVC-BSI in each study period (pre-, post-, and sustainment). The bacteria isolated were *Corynebacterium* species, methicillin-resistant *Staphylococcus epidermidis*, and *Cryptococcus neoformans* in an immunosuppressed patient; none were associated with a coinciding ESI/TI or nonintact dressing event. These cases yielded an incidence of 0.36 CVC-BSIs per 1000 patient-days (or catheter-days) in the preintervention period, 0.32 in the postintervention period, and 0.29 in the sustainment period.

## DISCUSSION

We identified a high rate of IV vancomycin use for suspected infections in a pediatric HD in an urban tertiary care children’s hospital. As shown in Figure 4, we expected a high use of IV vancomycin in the preintervention period; specifically, we noted significant overuse in July 2019, as indicated by the special cause event. Interventions, including staff and provider education, the identification of alternative antibiotics, and the implementation of a bedside RN exit-site scoring tool with a treatment-based algorithm, resulted in a downward shift in IV vancomycin use, with some variation. The subgroup of patients with nonintact dressings, in which the algorithm did not recommend antibiotic prophylaxis, significantly influenced the reduction in vancomycin use. Special cause events were noted, but they were associated with patients developing difficulties in treating ESI/TSI or CVC-BSI, which required the use of IV vancomycin.

The dialysis nurses quickly learned to use the exit-site scoring tool, as it was a similar tool used for PD catheter exit sites. The treatment-based algorithm was also well adopted by the APPs and physicians. However, we noted an increase in IV vancomycin doses during the sustainment period in September 2020 and June 2021 (Fig. 4), which was attributed to decreased adherence to the treatment-based algorithm, emphasizing the need for continued surveillance.

Despite the decrease in IV vancomycin use, patient safety was maintained, as the rates of CVC-BSIs did not increase in postintervention and sustainment periods. In the wake of the COVID-19 pandemic, when higher rates of CVC-BSIs were noted in adults and children with CVCs, we did not observe an increase in CVC-BSIs.^18,19^ Despite the Centers for Disease Control and Prevention recently recommending the use of bacitracin/neomycin/polymyxin B, we successfully reduced the CVC-BSI rate with the use of topical mupirocin.^20^ Although topical mupirocin is considered less effective than bacitracin/neomycin/polymyxin B in preventing catheter-related infections, we successfully reduced CVC-BSI rates with its use.^13^ Notably, we later changed our topical antibiotic to bacitracin/neomycin/polymyxin B.

Patients on HD are commonly prescribed vancomycin due to high rates of community MRSA prevalence, following the Infectious Diseases Society of America guidelines.^21^ We are the first HD center to report the volume of IV vancomycin use in pediatric patients with chronic HD. It is notable that the Standardizing Care to Improve Outcomes in Pediatric End-Stage Kidney Disease Collaborative publishes a BSI rate of 0.95 per 100 patient-months in those with any vascular access, but does not publish single-center IV vancomycin use or CVC-BSI rates.^4^ Furthermore, despite our reduced IV vancomycin use, our CVC-BSI rate remained competitive with the national benchmark (~0.87 per 100 patient-months in our sustainment period).

According to USRDS, outpatient expenditures for Medicare fee-for-service with ESRD were $12.8 billion. Although most of the cost is attributed to dialysis-related services ($10 billion), pharmacy-related costs are $1 million. Our QI initiative highlights that by practicing antibiotic stewardship, we can significantly reduce medication-related costs, especially those associated with infusion fees. The USRDS lacks granular data on outpatient dialysis unit costs, underscoring the significance of our study and the method of cost calculation.

There are a few limitations to our study. (1) Our QI team adapted the exit-site scoring of PD catheters.^11,12^ Although the subjective aspects of the tool could result in interobserver variability, there is a lack of validated tools to assess the exit site of tunneled CVCs. (2) We retrospectively audited the use of the exit-site scoring tool and repeatedly emphasized the importance of using the treatment protocol. However, the RN documentation of the exit-site score used in the postintervention period was only 66%. We also did not monitor the provider’s adherence to the treatment-based algorithm. (3) Despite having transparent data regarding medication charges, it is hard to know the true cost to the hospital system, as reimbursement through the ESRD Prospective Payment System was not included in our calculations. However, reducing dialysis-related medication use is a logical way to increase total reimbursement for ESRD care and promote value-based healthcare practices. Further cost transparency, including therapy drug monitoring costs, adverse event management costs, and alternative therapy opportunity costs, is necessary to reduce healthcare-related expenses.^22,23^

## CONCLUSIONS

We report a QI methodology that reduced the prescribing of IV vancomycin in a pediatric HD unit. We adopted an exit-site scoring tool for a CVC site assessment, which can be easily replicated in other HD units. With a stepwise implementation of staff education, standardization of CVC exit-site assessment, and institution of a treatment protocol incorporating topical and enteral antibiotics, we decreased our average vancomycin use from 24 doses per 1,000 patient-days to 5.1 doses per 1,000 patient-days (78%) in 1 year, without an increase in CVC-BSI. Furthermore, we reduced IV vancomycin-associated costs by 82% over the course of 1 year.

Future directions include collaborating with other pediatric dialysis centers and patient groups that use long-term CVCs to validate the exit-site scoring tool and reduce its subjectivity. Adopting a more frequent interdisciplinary team audit model to monitor IV vancomycin use in the unit would be beneficial.^24^ In addition, provider alerts through the EMR can help improve awareness and adherence to the exit-site scoring tool and treatment protocol algorithm.
